# Special Issue: The Role of Extracellular Matrix Proteins in Pathogenesis

**DOI:** 10.3390/ijms252413367

**Published:** 2024-12-13

**Authors:** Giuseppe Cappellano, Annalisa Chiocchetti, Davide Raineri

**Affiliations:** 1 Department of Health Sciences, Interdisciplinary Research Center of Autoimmune Diseases-IRCAD, University of Eastern Piedmont, 28100 Novara, Italy; giuseppe.cappellano@med.uniupo.it (G.C.); annalisa.chiocchetti@med.uniupo.it (A.C.); 2 Center for Translational Research on Autoimmune and Allergic Diseases, University of Eastern Piedmont, 28100 Novara, Italy

The extracellular matrix (ECM) serves as a complex network that regulates cellular behavior and maintains tissue architecture [1]. The ECM comprises several structural glycoproteins (collagens, proteoglycans/glycosaminoglycans, elastin, fibronectin, and laminins), as well as metalloproteinases (MMPs) and their inhibitors (TIMPs), which are involved in ECM remodeling. All these components, beyond providing structural support, play critical roles in cellular signaling and immune responses, impacting several physiological and pathological processes such as inflammation, fibrosis, tumor progression, and cardiovascular pathology. ECM proteins are increasingly being recognized as crucial players in the pathogenesis of several human diseases due to their roles in mechanical stability, cell signaling, and immune response modulation.

In this Special Issue, encompassing five original research articles and three reviews, we bring together diverse studies that explore the multifaceted contributions of ECM proteins to disease mechanisms, shedding light on their potential use as diagnostic biomarkers and therapeutic targets.

Mohamedi et al. investigated the protective functions of two ECM proteins, namely fibulin-2 and A disintegrin, and metalloproteinase with thrombospondin domains (ADAMTS-12) in lung inflammation and tumorigenesis. By generating a murine model double knockout for fibilin-2 and ADAMTS-12 genes, the authors found an exacerbated inflammatory response and an increased susceptibility to the development of lung tumors, highlighting their potential as therapeutic targets for mitigating inflammatory lung conditions and tumorigenesis [2].

Hulahan et al. investigated the impact of ECM alterations in the transition from ductal carcinoma in situ (DCIS) and invasive breast cancer (IBC) by using multiplexed spatial proteomics. Through this innovative approach, the study revealed distinct ECM profiles associated with DCIS and IBC; the authors identified specific collagen peptides that could discriminate between DICIS and IBC with a high specificity. The value of this study lies in its demonstration of how ECM profiling can enhance cancer diagnostics and monitoring, particularly by shedding light on the progression from DCIS to IBC and the role of the ECM in tumor invasion [3].

The effects of ECM modifications on mesenchymal stem cell (MSC) functions were shown by Komsa-Penkova et al. Among ECM proteins, collagen is essential for tissue repair and becomes structurally compromised through glycation, especially in conditions like diabetes and aging [4,5]. This study shows that glycated collagen can significantly alter MSC behavior, reducing cell spreading, adhesion, and cytoskeletal organization. These findings would suggest that non-enzymatic collagen modifications may impair MSC-based repair processes, highlighting the broader implications of ECM modifications in diseases marked by tissue degeneration [6].

In addition to structural molecules, the ECM also includes MMPs and their inhibitors, which are known as tissue inhibitors of metalloproteinases (TIMPs) [7]. MMPs are enzymes that are responsible for breaking down ECM components like collagen and elastin, playing a key role in tissue remodeling and repair. MMP activity is regulated by TIMPs in order to maintain ECM homeostasis [7].

In a rat model, Surówka et al. showed that long-term immunosuppressive treatments, usually used in transplanted subjects, may disrupt the balance between MMPs and their inhibitors (i.e., TIMPs), leading to structural abnormalities in cardiac tissue, such as collagen accumulation and cardiomyocyte hypertrophy. This study highlights the potential cardiovascular risks of chronic immunosuppression and the need to develop treatment regimens that maintain ECM balance to protect cardiac function in transplant patients [8].

A review of the literature on the potential of MMPs and TIMPs serving as prognostic biomarkers in head and neck squamous cell carcinoma (HNSCC) is provided by Fornieles et al., with a particular focus on their response to radiotherapy. Metastasis is a major challenge in HNSCC, and understanding how radiotherapy affects MMP and TIMP expression could aid in predicting patient outcomes. This review suggests the need for further research on MMPs and TIMPs as prognostic biomarkers, which could lead to more effective, personalized treatment strategies in managing HNSCC [9].

The role of proteoglycans in atherosclerosis and their effect on platelet function was investigated by Drysdale et al. The authors reported that proteoglycans were associated with different atherosclerotic plaque phenotypes. Biglycan and decorin were linked to ruptured plaques, while versican and hyaluronan were more prevalent in eroded ones. These findings suggest that these ECM components may inhibit thrombus formation and could serve as potential targets for developing plaque-specific anti-thrombotic therapies, highlighting the ECM’s critical role in regulating vascular pathology [10].

Moore et al. explored the impact of genetic factors on ECM integrity and their role in cardiovascular health. Traditionally associated with brittle cornea syndrome, mutations in Zinc finger protein 469 (ZNF469) and PR/SET Domain 5 (PRDM5) genes have recently been implicated in vascular diseases such as aneurysms and dissections [11]. By describing the effects of ZNF469 and PRDM5 mutations on ECM proteins like collagen, thrombospondin, and transforming growth factor beta 1 (TGF-β1), this study offers a novel perspective on the genetic regulation of ECM components, opening new avenues for understanding and potentially treating cardiovascular disorders [12].

Lastly, Chiu et al. elucidated recent findings on the molecular basis of the excessive proliferation and ECM production by orbital fibroblasts and the involvement of certain signaling pathways, leading to tissue expansion/remodeling and fibrosis in Graves’ ophthalmopathy [13]. The authors suggest that broadening the concepts of the ECM and tissue fibrosis will help in the development of new therapeutic targets, including multiple antioxidants and antifibrotic agents.

In conclusion, these studies underscore the pivotal role of ECM proteins in the pathogenesis of a wide array of diseases. Future research aimed at unraveling the complex interactions within the ECM will not only deepen our understanding of disease mechanisms, but also reveal novel therapeutic targets. By developing treatments that specifically modulate ECM proteins and pathways, we can make significant strides in mitigating disease progression, improving diagnostics, and enhancing patient outcomes across various medical fields.
